# A mechanistic kinetic description of lactate dehydrogenase elucidating cancer diagnosis and inhibitor evaluation

**DOI:** 10.1080/14756366.2016.1275606

**Published:** 2017-01-24

**Authors:** Peifeng Tang, Jianlin Xu, Christopher L. Oliveira, Zheng Jian Li, Shijie Liu

**Affiliations:** a Department of Paper and Bioprocess Engineering, SUNY ESF, Syracuse, NY, USA;; b Biologics Process Development, Global Manufacturing and Supply, Bristol-Myers Squibb Company, Devens, MA, USA

**Keywords:** Cancer diagnosis, inhibitor evaluation, kinetic model, lactate dehydrogenase, oligomeric enzyme

## Abstract

As a key enzyme for glycolysis, lactate dehydrogenase (LDH) remains as a topic of great interest in cancer study. Though a number of kinetic models have been applied to describe the dynamic behavior of LDH, few can reflect its actual mechanism, making it difficult to explain the observed substrate and competitor inhibitions at wide concentration ranges. A novel mechanistic kinetic model is developed based on the enzymatic processes and the interactive properties of LDH. Better kinetic simulation as well as new enzyme interactivity information and kinetic properties extracted from published articles via the novel model was presented. Case studies were presented to a comprehensive understanding of the effect of temperature, substrate, and inhibitor on LDH kinetic activities for promising application in cancer diagnosis, inhibitor evaluation, and adequate drug dosage prediction.

## Introduction

**Figure 1.**
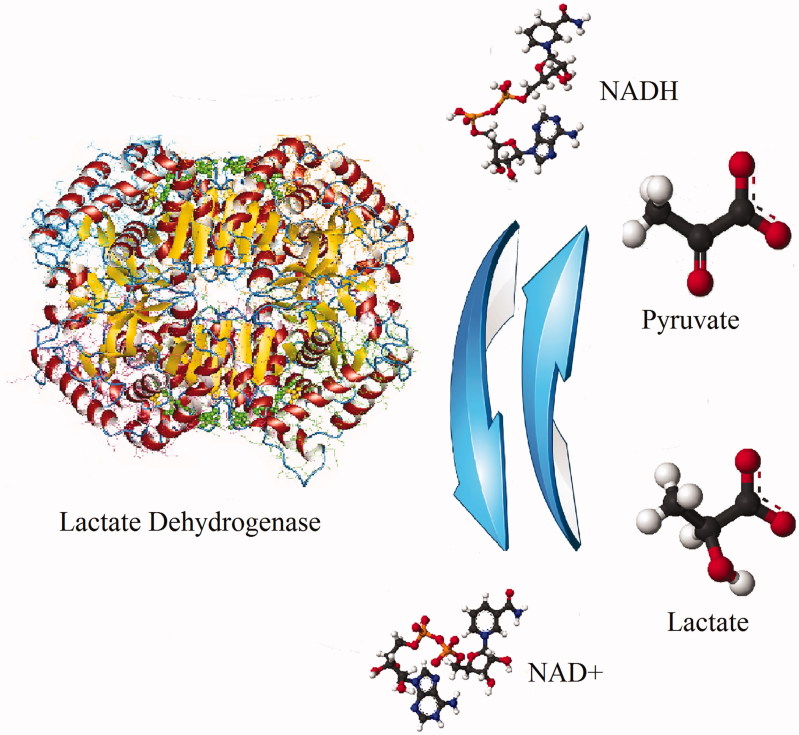
Scheme of mutual transformation of pyruvate and lactate catalyzed by lactate dehydrogenase.

Lactate dehydrogenase (LDH) is an essential enzyme in nearly all living cells^1^^,^^2^, which catalyzes the mutual transformation between pyruvate and lactate, associated with NADH and NAD + interconversion^3^ (Figure 1). Because cancer cells heavily rely on aerobic glycolysis to support their growth, LDH comes to be an emerging anticancer target for cancer diagnosis and treatment^4^. Besides the enzyme level and catalytic mechanism, its kinetic properties have also attracted wide interests^5^^,^^6^. From 1997 to 2016, approximately 1625 publications are found as LDH kinetic related, based on our query and manual filtration on a peer-reviewed literature database named as Scopus^®^.

**Figure 2.**
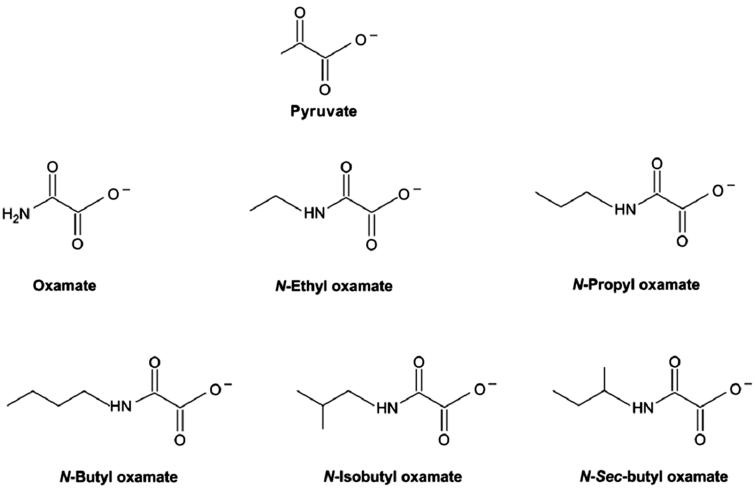
Structures of pyruvate and oxamate derived inhibitors^37^.

To inhibit the glycolysis within cancer cells, hundreds of small molecules are under study to reduce LDH activity. One type of inhibitor candidates are molecules that have similar chemical structures as pyruvate and are able to competitively associate on the substrate domain. Oxamate derivatives (Figure 2), e.g. are one type of model inhibitors^7^. These molecules are able to seize the available substrate-binding sites and further inhibit substrate binding and reactivity. Meanwhile, tumor cells respond differently from normal cells to temperature changes^8^. Therefore, a universal method to quantitatively describe the kinetic properties of LDH and evaluate the effect of inhibitors and temperature can be beneficial for cancer study.

As a tetramer existing in most physiological environments^9^^,^^10^, one LDH molecule has four subdomains, and thus, is able to bind up to four substrate molecules and four coenzyme molecules. The binding of one or more substrate and coenzyme molecules regulates the oligomeric enzyme protein folding, leading to dynamic enzyme activity change. Thus, LDH is an interactive enzyme known to possess allosteric properties^11^^,^^12^.

Several kinetic models have been applied to describe LDH performance. However, few are able to elucidate the enzymatic process mechanistically. Known models include Michaelis–Menten (M–M) Equation, Hill Equation^13^^–^16, Binding Model of Alberty^17^^,^^18^, Monod–Wyman–Changeux (MWC) Model^11^^,^^19^, and empirical models derived from software of JMP^18^. The deficiency of these models is mainly the failure in taking a comprehensive consideration of the complex interactive processes and allosteric properties of the oligomeric enzyme.

The objective of this paper is to develop a mechanistic kinetic model based on the interactive enzyme properties that is capable of describing temperature, substrate and inhibitor effect. It aims at better kinetic simulation of the enzymatic process, a comprehensive understanding of the enzyme behavior in complex conditions, as well as the promising applications in cancer diagnosis, inhibitor evaluation and adequate medication dosage.

## Model development

Due to the interactive nature, an oligomeric enzyme exhibits unique kinetic properties at each successive binding. Thus, a mechanistic kinetic model needs to take all the information of enzyme interactive regulations caused by sequential coenzyme, substrate, and inhibitor binding into consideration. In order to better understand the enzyme kinetic properties and simplify the mathematic formula, this study focuses on the initial reaction rates. An isoenzyme consisted of identical subunits is used as the modeling example. Therefore, the binding and dissociation processes can be expressed as

(1-a) $$E_{\text{n}}S_{\text{i}-\text{1}}+S\to E_{\text{n}}S_{\text{i}},\to \;r_{+\text{ni}}=\;\left(n-i+\text{1}\right)k_{\text{ni}+}\left[E_{\text{n}}S_{\text{i}-\text{1}}\right]\left[S\right]$$

(1-b) $$E_{\text{n}}S_{\text{i}}\to E_{\text{n}}S_{\text{i}-\text{1}}+S,\to \;r_{-\text{ni}}=ik_{\text{ni}-}\left[E_{\text{n}}S_{\text{i}}\right]$$

where, *n* is the total number of available binding sites of the oligomeric enzyme *E*_n_, *S* denotes for the binding substrate, *E*_n_*S*_i_ denotes for one enzyme molecule bound with *i* substrate molecules, *k*_ni+ _ is the binding rate constant for substrate molecule on each of the (*n*−*i* + 1) vacant sites, and *k*_ni−_ is the dissociation of a substrate molecule from each of the *i* bound substrate molecules.

It is assumed that the binding is much faster than the reaction. Thus, thermodynamic equilibrium applies to substrate binding on all the sites, which is

(2) $$[E_{n}S_{i}]=\frac{\left(n-i+1)k_{ni+}[E_{n}S_{i-1}][S\right]}{ik_{ni-}}=\left(\begin{array}{c} n\\ i \end{array}\right)\frac{[E_{n}]{\left[S\right]}^{i}}{K_{n}\prod_{j=1}^{i-1}\left(\alpha_{nj}K_{n}\right)}$$

where

(3-a) $$\left(\begin{array}{c} n\\ i \end{array}\right)=\frac{n\cdot (n-1)\cdot \cdot \cdot (n-i+1)}{1\cdot 2\cdot \cdot \cdot i}$$

(3-b) $$K_{n}=\frac{k_{n1-}}{k_{n1+}}$$

(3-c) $$\alpha_{\mathrm{ni}-1}=\frac{k_{\mathrm{ni}-}}{k_{\mathrm{ni}+}}{\left(\frac{k_{n1-}}{k_{n1+}}\right)}^{-1}$$

Considering the mass balance of the enzyme leads to

(4) $$E_{n}=[E_{n}]+\sum_{i=1}^{n}\left[E_{n}S_{i}\right]=[E_{n}]+\frac{\left[E_{n}][S\right]}{K_{n}}\sum_{i=1}^{n}\left(\begin{array}{c} n\\ i \end{array}\right)\frac{{\left[S\right]}^{i-1}}{\prod_{j=1}^{i-1}\left(\alpha_{nj}K_{n}\right)}$$

Substituting Equation (4) into (2) yields

(5) $$[E_{n}S_{i}]=\frac{E_{n}\left(\begin{array}{c} n\\ i \end{array}\right)\frac{{\left[S\right]}^{i}}{\prod_{j=1}^{i-1}\left(\alpha_{nj}K_{n}\right)}}{K_{n}+[S]\sum_{k=1}^{n}\left(\begin{array}{c} n\\ k \end{array}\right)\frac{{\left[S\right]}^{k-1}}{\prod_{j=1}^{k-1}\left(\alpha_{nj}K_{n}\right)}}$$

The overall catalytic reaction rate for an oligomeric enzyme with *n*-reactive binding sites, *r_Pn_*, is then given by

(6) $$r_{\mathrm{Pn}}=k_{n}[E_{n}S]+k_{n}\sum_{i=2}^{n}i\beta_{\mathrm{ni}-1}[E_{n}S_{i}]$$

where, *β* is the coefficient indicating the interactive effect on reactivity.

## Results

### LDH saturated with coenzyme

**Figure 3.**
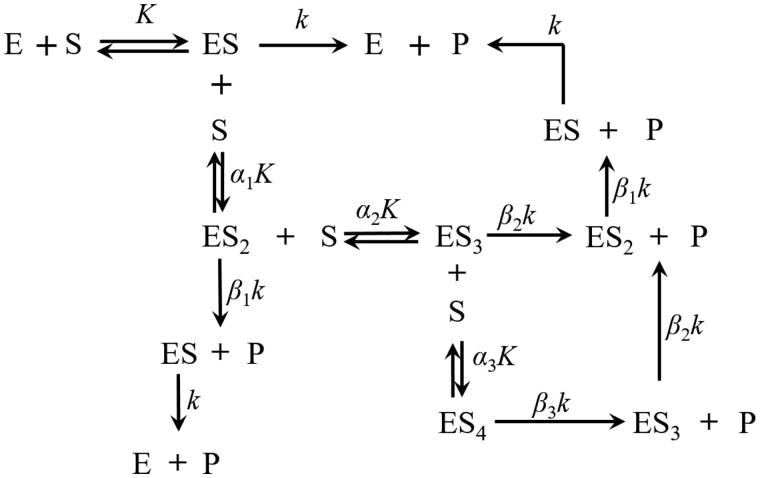
Sequential substrate binding and reaction processes on LDH at the coenzyme saturation condition.

LDH, as an interactive tetrameric enzyme, follows the general mechanistic kinetic principles expressed above. In studies conducted to examine the substrate (pyruvate or lactate) effect on LDH kinetic activities, an excess of coenzyme (NAD^+ ^or NADH) is generally added to saturate and fully activate the enzyme^20^^,^^21^. Based on the reaction mechanism (Figure 3) and Equation (1) to (6), the reaction rate can be expressed as

(7) $$r=4\frac{\mathrm{kE}[S]}{\mathrm{Kf}}\left(1+\frac{3\beta_{1}[S]}{\alpha_{1}K}+\frac{3\beta_{2}[S]{}^{2}}{\alpha_{1}\alpha_{2}K^{2}}+\frac{\beta_{3}[S]{}^{3}}{\alpha_{1}\alpha_{2}\alpha_{3}K^{3}}\right)$$

where, *E* is the coenzyme saturated LDH isoenzyme, which is the same as *E*_4_. *f* is defined as

(8) $$f=1+\frac{4[S]}{K}+\frac{6[S]{}^{2}}{\alpha_{1}K^{2}}+\frac{4[S]{}^{3}}{\alpha_{1}\alpha_{2}K^{3}}+\frac{[S]{}^{4}}{\alpha_{1}\alpha_{2}\alpha_{3}K^{4}}$$

While it is conceivable to have the affinities vary extensively during the subsequent binding, there could be a pattern of affinity change. In this case, the cooperative effect is significantly simplified. If we let

(9-a) $$\alpha_{1}=\alpha_{2}=\alpha_{3}=\alpha$$

(9-b) $$\beta_{1}=\beta_{2}=\beta_{3}=\beta$$

The model now signifies that the successive binding approaches the same incremental proportional affinity change.

Then, Equation (7) can be simplified to

(10) $$r=\frac{4\mathrm{kE}[S]}{K}\frac{1-\beta +\beta {\left(1+\frac{\left[S\right]}{\alpha K}\right)}^{3}}{1-\alpha +\alpha {\left(1+\frac{\left[S\right]}{\alpha K}\right)}^{4}}$$

Specifically, at a low substrate level, Equation (10) can be further simplified to

(11) $$r=\frac{\mathrm{kE}[S]}{\frac{K}{4}+[S]}$$

Equation (11) is the familiar M-M formula. Reaching this result after applying the aforementioned conditions explains how the M-M model can approximately simulate the kinetic process at low substrate concentrations. Equation (10) is in agreement with the conventional M-M model but is applicable outside of the limited condition.

Generally, α < 1 is favorable for subsequent substrate binding; β > 1 indicates that subsequent binding accelerates the on-site reaction.

### LDH competitive effector binding model

**Figure 4.**
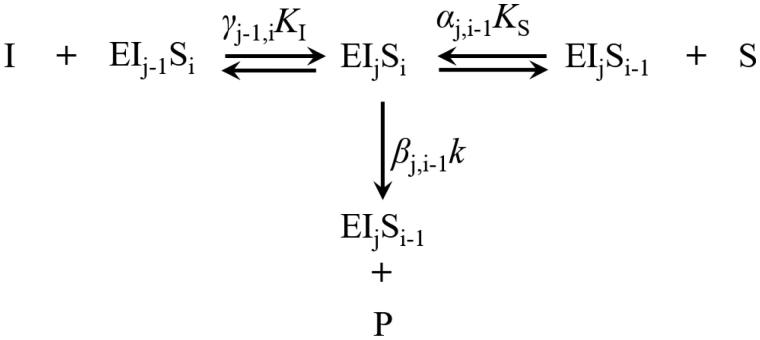
Typical substrate and inhibitor binding and reaction processes. (1 ≤ i, j, i + j ≤ 4). S, P, and I are the substrate, product, and competitive effector, respectively; *α*, *β,* and *γ* represent the kinetic constants change due to interactive effects.

Inhibitors, such as oxamate derived molecules (Figure 2), can seize the LDH substrate binding sites (Figure 4) and reduce the enzyme activity. Therefore, at the coenzyme saturated condition, the process with competitive effectors can be expressed as

(12) $$r=\sum_{i=1}^{4}\sum_{j=0}^{4-i}i\beta_{j,i-1}k[{\mathrm{EI}}_{j}S_{i}]$$

(13-a) $$\left[{\mathrm{EI}}_{j}]=\frac{\left(5-j)[{\mathrm{EI}}_{j-1}][I\right]}{j\gamma_{j-1,0}K_{I}}\;\;(1\leq j\leq 4\right)$$

(13-b) $$\left[{\mathrm{ES}}_{i}]=\frac{\left(5-i)[{\mathrm{ES}}_{i-1}][S\right]}{i\alpha_{0,i-1}K_{S}}\;\;(1\leq i\leq 4\right)$$

(13-c) $$\left[{\mathrm{EI}}_{j}S_{i}]=\frac{1}{2}\left\{\frac{\left(5-j-i\right)[{\mathrm{EI}}_{j}S_{i-1}][S]}{i\alpha_{j,i-1}K_{S}}+\frac{\left(5-j-i\right)[{\mathrm{EI}}_{j-1}S_{i}][I]}{j\gamma_{j-1,i}K_{I}}\right\}\;\;\times (1\leq i,j,i+j\leq 4\right)$$

(14) $$E=\sum_{i=0}^{4}\sum_{j=0}^{4-i}[{\mathrm{EI}}_{j}S_{i}]$$

where, *S* and *I* are the substrate and inhibitor, respectively; *k* is the reaction rate constant of the first binding substrate without inhibitor; *K* is the saturation coefficient of the first binding of substrate (*K_S_*) or inhibitor (*K_I_*); *α, β* and *γ* are the interactive coefficients. The subscripts *i* and *j* associated with *I* and *S* indicate the number of substrate and inhibitor molecules bound to the enzyme complex, respectively; *α* and *γ* indicate the interactive binding effect on subsequent substrate and inhibitor, respectively; *β* indicates the interactive effect on reactivity.

Following a similar assumption as Equations (9-a) and (9-b), we let

(15-a) $$\alpha_{m,n}=\alpha \;\;(1\leq m+n\leq 3)$$

(15-b) $$\beta_{0,m}=\beta_{S}\;\;(1\leq m\leq 3)$$

(15-c) $$\beta_{n,m}=\beta_{\mathrm{IS}}\;\;(1\leq n,n+m\leq 3)$$

(15-d) $$\gamma_{n,m}=\gamma \;\;(1\leq m+n\leq 3)$$

where, subscript *_S_* indicates no inhibitor bound on the enzyme molecule and the interactive effect is only caused by subsequent substrate binding; subscript *_IS_* indicates that the interactive effect is caused by both inhibitor and substrate molecules bound on the enzyme complex.

Substituting Equations Equation (13), (14), and (15) into (12) leads to

(16) $$r=\frac{4\mathrm{kE}[S]}{K_{S}F}{\{1-\beta_{S}+\beta_{S}\left(1+\frac{\left[S\right]}{\alpha K_{S}}\right)}^{3}+\frac{\beta_{\mathrm{IS}}[I]}{\gamma K_{I}}(\frac{3}{2}+\frac{3\lambda }{2\alpha }+\frac{3[I]}{4\gamma K_{I}}\left(1+\frac{3\gamma }{\alpha }\right)\;+\frac{[I]{}^{2}}{8{\left(\gamma K_{I}\right)}^{2}}\left(1+\frac{7\gamma }{\alpha }\right)+\frac{\left[S\right]}{\alpha K_{S}}\left(\frac{9}{2}+\frac{3\lambda }{2\alpha }+\frac{3[S]}{8\alpha K_{S}}\left(7+\frac{\gamma }{\alpha }\right)+\frac{3[I]}{2\lambda K_{I}}\left(1+\frac{\gamma }{\alpha }\right)\right))\}$$

where,

(17) $$F=1-\alpha -\gamma +\alpha {\left(1+\frac{\left[S\right]}{\alpha K_{S}}\right)}^{4}+\gamma {\left(1+\frac{\left[I\right]}{\gamma K_{I}}\right)}^{4}\;+\frac{\left[I][S\right]}{K_{I}K_{S}}\{\frac{6}{\alpha }+\frac{6}{\lambda }+\frac{3[I]}{\gamma K_{I}}\left(\frac{3}{\alpha }+\frac{1}{\gamma }\right)+\frac{[I]{}^{2}}{2{\left(\gamma K_{I}\right)}^{2}}\left(\frac{7}{\alpha }+\frac{1}{\gamma }\right)\;+\frac{3[I][S]}{\gamma K_{I}\alpha K_{S}}\left(\frac{1}{\alpha }+\frac{1}{\gamma }\right)+\frac{[S]{}^{2}}{2{\left(\alpha K_{S}\right)}^{2}}\left(\frac{7}{\gamma }+\frac{1}{\alpha }\right)\}$$

Specifically, at a low inhibitor and substrate level, higher order items are negligible; therefore, Equation (16) can be simplified to

(18) $$r=4\mathrm{kE}\frac{[S]\left\{1+\frac{3\beta_{\mathrm{IS}}[I]}{2K_{I}}\left(\frac{1}{\gamma }+\frac{1}{\alpha }\right)\right\}}{K_{S}+4[S]+\frac{4K_{S}[I]}{K_{I}}}$$

Equation (18) is mathematically similar to the binding model of Alberty^17^^,^^18^^,^^22^. This explains the applicability of the conventional binding model of Alberty for LDH, under the low substrate and inhibitor condition.

At low inhibitor and moderate substrate concentrations, [*I*] items are negligible, then Equation (16) can be simplified to

(19) $$r=\frac{4\mathrm{kE}[S]}{K_{S}}\frac{1-\beta_{S}+\beta_{S}{\left(1+\frac{\left[S\right]}{\alpha K_{S}}\right)}^{3}}{1-\alpha +\alpha {\left(1+\frac{\left[S\right]}{\alpha K_{S}}\right)}^{4}}$$

Equation (19) is identical to Equation (10), which demonstrates that Equation (16) is a more general equation for the initial rate of LDH catalyzed reactions.

## Discussion

### Substrate concentration and temperature effects

To study the substrate concentration and temperature effects, human LDH type 1 (hLDH-1) was used as an example. Three data sets were selected from two publications, reported by Vesell^23^ and Gubernieva et al.^24^, for the initial catalytic rates of hLDH-1 in converting pyruvate to lactate. The isoenzymes were extracted from human heart, psoas muscle, liver, or pancreas tissue within 12 h of death. The experiment was controlled at 20, 25, and 37 °C, respectively. The isoenzymes were pre-saturated by NADH before initial rates were measured. The initial reaction rates were measured with different levels of pyruvate by a spectrometer at 340 nm. The data were selected from these publications and reanalyzed using Equation (10).

**Figure 5.**
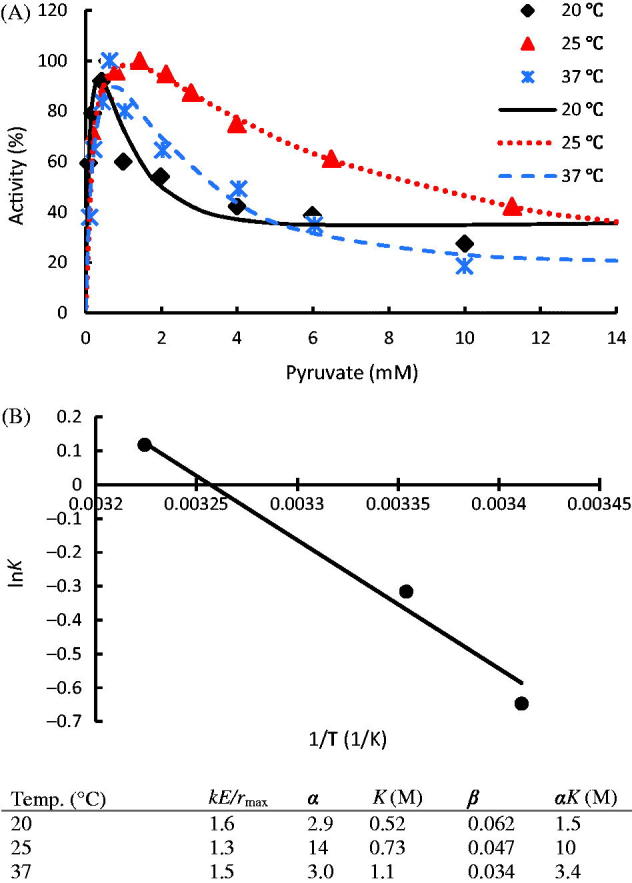
(A) Temperature effect on the activity of hLDH-1 with data taken from publications^23^^,^^24^; (B) Van’t Hoff Equation simulation of hLDH-1 *K* values. hLDH-1 kinetic parameters at different reaction conditions are listed in the table. The dots denote the experimental data and the lines represent the simulation results.

Enzyme hLDH-1 exhibited sensitive responses to temperature (Figure 5). This isoenzyme reported significant substrate inhibition when pyruvate concentration is higher than 1 mM. The calculated β < 1 indicates that the subsequent substrate binding reduced the reactivity. Thus, a higher ratio of [ES*_i_*]/E (*i* >1) could lower LDH activity at a high pyruvate concentration. A smaller α indicates enhanced binding on subsequent sites. Therefore, at 37 °C, reporting small *α* and *β* values, could be the preferable choice for suppressing the activity of hLDH-1.

Equation (10) can be applied to predict the substrate concentration when the activity reaches peak value. If we let

(20) $$\frac{\mathrm{dr}}{d[S]}=0$$

the theoretical substrate concentrations to reach the peak activity can be calculated as 0.4, 1.1, and 0.7 mM at 20, 25, and 37 °C, respectively, which agree with observed values in the experiments (Figure 5).

Besides explaining the interactive properties and simulating the kinetic observations, Equation (10) helped to reach a comprehensive understanding of temperature on pyruvate binding, which was not reported elsewhere. For a robust enzyme, if its structure is not sensitive to temperature changes, the binding saturation coefficient should approximately follow the Van’t Hoff’s Equation^25^ as

(21) $$K=\frac{k_{-}}{k_{+}}=\frac{k_{0-}\exp\left(-\frac{E_{a-}}{\mathrm{RT}}\right)}{k_{0+}\exp\left(-\frac{E_{a+}}{\mathrm{RT}}\right)}=K_{0}\exp\left(-\frac{\Delta E_{a}}{\mathrm{RT}}\right)$$

where, Δ*E_a_* is the activation energy difference between the backward and forward reaction or the heat of binding, *R* is the ideal gas constant, *T* is temperature (K); *K*_0_ is a constant.

The approximately linear regression indicates that hLDH-1 is a relatively robust enzyme within the studied temperature range of 20 ∼ 37 °C (Figure 5B). The calculated Δ*E_a_* was 31.5 kJ/mol. The relatively integral enzyme structure indicates that the hLDH-1 subunits are relatively rigid. The initial binding affinity at other temperatures within the studied temperature range can thus be predicted via this model.

### Cancer diagnosis

Cancer is one of the global leading causes of death^26^. Abnormal LDH kinetic properties and concentrations have been observed for tumor cells in comparison to normal cells^27^^,^^28^. Currently, LDH is a useful marker for diagnosing cancer due to its role in the final step of the aerobic glycolysis^29^.

One set of data from experiments conducted by Talaiezadeh6 was reprocessed with current model to provide insight on its behavior. In the work by Talaiezadeh et al., LDH isoenzymes were extracted and purified from normal and malignant human breast tissues. The enzymes were dissolved in a pH 8 buffer solution. The experiments were conducted similarly to those used in the Section “Substrate concentration and temperature effects”.

**Figure 6.**
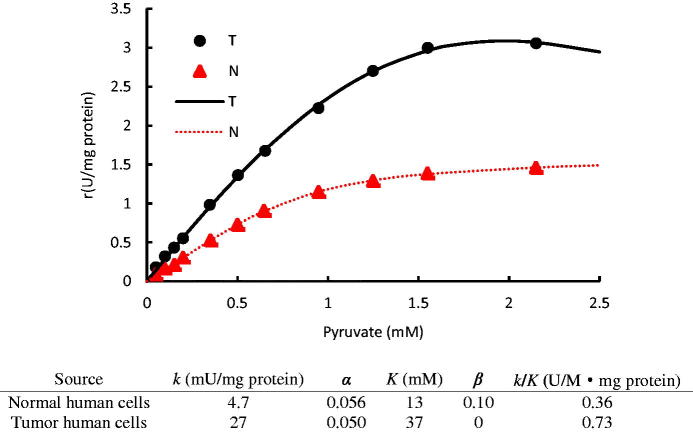
The initial rates of LDH from tumor human breast cells (T) and normal human breast cells (N) vary with substrate concentrations in conversion of pyruvate to lactate; The data were taken from the publication^6^; Kinetic parameters of different LDH isoenzymes are listed in the table. The dots denote the experimental data and the lines represent the simulation results.

Figure 6 shows that LDH from tumor human breast cells (T-hLDH) exhibited an enhanced catalytic activity as compared to the isoenzyme from normal human breast cells (N-hLDH). This is confirmed by the *k* values of T-LDH, which was five-fold larger than that of N-LDH. The small *α* values of both LDH isoenzymes indicate that subsequent substrate binding was favored by the cooperative effect. However, this cooperative effect inhibited the enzymatic activity, indicated by the *β* < 1. The smaller *β* value of T-LDH than that of N-LDH indicates that T-LDH is a more substrate sensitive isoenzyme than N-LDH. Since dynamic intracellular pyruvate concentration always remains low in mammalian cells^30^^,^^31^, a quantitative analysis of *k*/*K* showed that the reaction within tumor cells could be potentially two-fold of that in normal cells at very low pyruvate concentrations (Figure 6). This explains the abnormal high glycolysis metabolic activity observed in tumor cells^32^^,^^33^.

While N-LDH still retained a reduced activity (*β* = 0.1), T-LDH became inactive (*β* = 0) when more than one substrate molecule was bound on it. Therefore, molecules which have similar structures as pyruvate may strongly inhibit T-LDH activity, while have lesser effects on N-LDH.

### Inhibitor evaluation and dosage prediction

Because most tumor cells heavily rely on an enhanced glycolysis activity in converting glucose to lactate to maintain metabolic requirements^34^, studies have been focused on those inhibitors, which can significantly reduce LDH activity for potential cancer treatment^35^^,^^36^.

The experiment data were reported by Rodriguez-Paez^37^. Initial rates of pyruvate conversion to lactate catalyzed by mouse LDH were measured with different additions of N-propyl oxamate. The experiments were conducted similarly as the case studies utilized in the previous sections.

**Figure 7.**
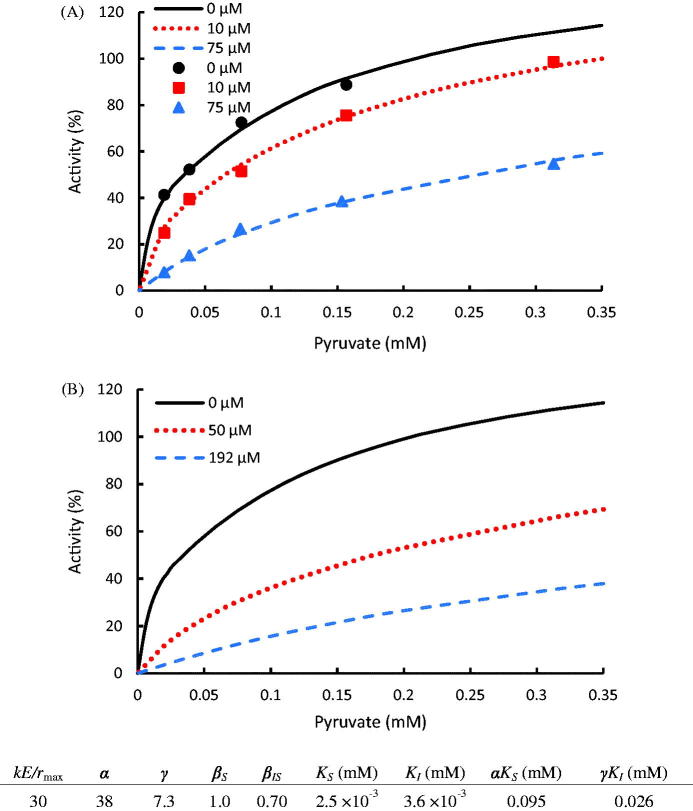
(A) Inhibition of N-propyl oxamate on LDH for the conversion of pyruvate to lactate with experimental data taken from the publication^37^. (B) Kinetic prediction of N-propyl oxamate required to inhibit LDH activity at 50 and 25% at 0.2 mM pyruvate concentration. The dots denote the experimental data and the lines represent the computing results.

The data were reprocessed via Equation (16) and shown in Figure 7. One set of parameters was able to elucidate the kinetics at various inhibitor and substrate conditions. Thus, quantitative standards can be built up to characterize the effect of an inhibitor regardless of its dosage. Typically, small *γ*, *K_I_*, and *β_IS_* values, which indicate high binding competitiveness and reduced reactivity, are preferred for a high performance inhibitor. In this case study, the calculated *α*, *γ* > 1 indicates that both pyruvate and N-propyl oxamate are retarding ligands, inhibiting subsequent substrate and inhibitor binding on the enzyme. N-propyl Oxamate exhibited lower binding affinity than pyruvate at low concentrations (*K_I _*>*_ _K_S_*), while its binding affinity exceeded pyruvate at high concentrations (*γK_I _*< *αK_S_*). Substrate inhibition was not observed from this experiment (*β_S_* = 1.0). However, when both substrate and inhibitor molecules were bound, the LDH activity could be reduced by the substrate-inhibitor cooperation (*β_IS_* = 0.7). Therefore, oxamate inhibited LDH activity not only by seizing the substrate binding domains but also by reducing the reactivity. This makes N-propyl oxamate a qualified inhibitor reducing LDH activity within wide substrate concentration ranges.

Besides the mechanistic understanding of the enzyme properties, this model also serves for adequate inhibitor dosage study. An accurate inhibitor dosage to reach a target LDH activity can be predicted via this model instead of testing in the lab. For instance, based on the disease diagnosis study (Figure 6). The enzymatic activity within a normal cell is 50% of a tumor cell around the pyruvate concentration of 0.2 mM, which is a typical level within cells^38^. Using Equation (16), it can be calculated that 50 μM of N-propyl oxamate can successfully halve the activity of LDH, while 192 μM can further halve it (Figure 7(B)). This provides the basis to set up adequate inhibitor or drug doses, avoiding any insufficient or excessive dosage.

Overall, this model builds up uniform standards to evaluate different kinds of inhibitors by comparing *γ*, *K_I_*, and *β_IS_* values. It is able to use one set of parameters to mechanistically evaluate the inhibitors regardless of their concentrations, instead of using classical M-M model to get different sets of kinetic parameters at various inhibitor levels. Besides, it can compute a theoretical inhibitor dosage to reach a target inhibitory effect, which may reduce the pharmaceutical research cost during the clinical or preclinical stage.

## Conclusions

In this paper, a new mechanistic kinetic model was developed to explain the kinetic properties and dynamic behaviors of oligomeric enzymes, such as LDH, based on the interactive nature of enzyme-ligand bindings. The effects of temperature, substrate concentrations, and competitive effectors were analyzed mechanistically with case studies. The kinetic information extracted from the new models provided a theoretical understanding of LDH, and was able to evaluate inhibitor effect with uniform standards and predict the kinetics at different substrate and inhibitor levels. This may promote further advancements in disease diagnosis, enzyme activity control, drug evaluation as well as adequate medication dosage.
